# Effect of Parenting Style, attachment to parents and self-compassion on the occurrence and recovery motivation for non-suicidal self-injury in adolescents

**DOI:** 10.3389/fpsyg.2024.1408396

**Published:** 2024-09-03

**Authors:** Pei Liu, Jianbo Liu, Yi Li, Jianping Lu

**Affiliations:** ^1^State Key Laboratory of Chemical Oncogenomics, Guangdong Provincial Key Laboratory of Chemical Genomics, Peking University Shenzhen Graduate School, Shenzhen, China; ^2^Department of Child and Adolescent Psychiatry, Shenzhen Kangning Hospital, Shenzhen Institute of Mental Health, Shenzhen Mental Health Center, Shenzhen Clinical Research Center for Mental Disorders, Shenzhen, China

**Keywords:** non-suicidal self-injury, recovery motivation, self-compassion, adolescent, attachment to parents

## Abstract

**Background:**

Non-suicidal self-injury (NSSI) is a worldwide mental health problem that deserves thorough investigation. This study aims to explore the effect of parenting styles, attachment to parents, and self-compassion on the occurrence of non-suicidal self-injury (NSSI) behavior in adolescents and whether these factors influence their recovery motivation.

**Methods:**

A total of 132 adolescents who had engaged in NSSI within the last year and 72 adolescents who had never engaged in NSSI were recruited from the Shenzhen Kangning Hospital and from primary and secondary schools and communities. Differences in the Hamilton Depression Scale (HAMD), Inventory of Parent and Peer Attachment (IPPA), Egma Minn av. Bardndosnauppforstran (EMBU), and Self-Compassion Scale (SCS) of participants were compared. A binary logistic model was established to measure the odds ratios of these variables on the occurrence of NSSI. In the NSSI adolescent sample, separate binary logistic models were created with NSSI impulse inhibition, NSSI resistance activity, and NSSI recovery motivation as dependent variables and with parenting styles, attachment to parents, and self-compassion as independent variables.

**Results:**

Compared with adolescents with no NSSI behavior, those who had engaged in NSSI within the past year had higher scores on the HAMD, as well as higher EMBU-negative father parental behavior (punishment, excessive interference, rejection, and overprotection), EMBU-negative mother parental behavior (excessive interference, rejection, and punishment), and SCS negative self-compassion scores. Moreover, adolescents with NSSI had lower EMBU-father emotional warmth, EMBU-mother emotional warmth, IPPA-attachment to father, IPPA-attachment to mother, and SCS positive self-compassion scores. Current depressive symptoms and maternal punishment are risk factors for NSSI in adolescents, while positive self-compassion was a protective factor. Positive self-compassion can positively predict NSSI impulse inhibition, NSSI resistance activity, and NSSI recovery motivation. However, we unexpectedly found that the father’s emotional warmth negatively predicts NSSI resistance activity.

**Conclusion:**

This study found that positive self-compassion has a significant impact on the prevention of and recovery from NSSI behavior in adolescents.

## Introduction

Non-suicidal self-injury (NSSI) is defined as the direct and intentional injury to one’s body without the intention to die (Nock, 2010). The global prevalence of NSSI of a non-clinical sample over a lifetime was around 22.0% (Xiao et al., 2022); among the Chinese population, the total average estimated lifetime prevalence of NSSI was 24.7%, with averages of 26.5% in females and 27.7% among males (Qu et al., 2023). The most common NSSI methods are cutting, but scratching and biting are also prevalent (Qu et al., 2023). The NSSI frequency increases in young adolescents, remains stable in middle adolescents, and decreases in older adolescent (De Luca et al., 2023). Adolescents who engaged in NSSI were also more likely to engage high-risk behaviors, including substance abuse and risky sexual behavior, as well as to demonstrate negative mental health symptoms such as depression, anxiety, stress, and borderline personality disorder (Cipriano et al., 2017). In the DSM-5, NSSI is proposed as a “condition for further study,” with diagnostic criteria that include engaging in self-harm on five or more days within the past year without suicidal intent and where these behaviors serve a particular function, such as alleviating negative feelings or thoughts, resolving interpersonal difficulties, or inducing a positive emotional state. These behaviors have particular precursors, are not socially sanctioned, result in impairment across multiple dimensions, and cannot be better explained by another mental disorder (American Psychiatric Association, 2013). Relevant clinical research includes studies with subjects who meet the DSM-5 criteria, as well as those that explore NSSI behaviors occurring more than a year prior or subthreshold NSSI behaviors (i.e., fewer than 5 days of self-harm within a year).

NSSI among children and adolescents can be viewed as an act of hostility, aggression, and anger toward oneself that could stem from a lack of self-compassion. The development of self-compassion in adolescents is linked to their positive attachment to their parents, which in turn originate from effective parenting styles. Parenting styles, children’s attachment to their parents, and self-compassion are all related to NSSI behaviors in adolescents. Clinical research has indicated that parenting styles that result in a high risk of NSSI for children include high father rejection, mother rejection (Liu et al., 2020; Zhu et al., 2020; Ying et al., 2021), lower mother emotional warmth (Tschan et al., 2015; Liu et al., 2020), mother overprotection (Liu et al., 2020; Ying et al., 2021), authoritarian parenting styles (Liu et al., 2022), harsh or punitive parenting (Victor et al., 2019; Liu et al., 2021; Gu et al., 2023), lack of monitoring children (Victor et al., 2019), and highly expressed emotion-criticism (James and Gibb, 2019). Several studies show that adolescents who engage in NSSI have lower scores on parental attachment characteristics than the control group (Jiang et al., 2017; Santangelo et al., 2017; Bahali et al., 2023). Consistent with this, adolescents’ self-assessed attachment to their mothers decreased after one event of self-injury (Koenig et al., 2021). Self-compassion refers to being supportive toward oneself when experiencing suffering or pain. Most of the current research on NSSI and self-compassion is cross-sectional. These studies found that a higher level of positive self-compassion correlated to fewer NSSI behaviors. In comparison, a higher level of negative self-compassion correlated to higher NSSI frequency (Jiang et al., 2017; Liu et al., 2023). Meanwhile, evidence has shown that an individual’s level of self-compassion might reduce the negative influence of many risk factors for NSSI behaviors; such factors include emotional neglect (Erol and Inozu, 2023), childhood trauma (Wu et al., 2023), and peer bullying (Jiang et al., 2016).

Parenting style, parent–child attachment, and adolescent self-compassion are all highly correlated with each other. When parents adopt better parenting strategies, such as being able to respond better to their children’s emotions, children will develop a more secure attachment to their parents, which will in turn cultivate adolescents’ ability to be self-compassionate (Nasika et al., 2023). Previous cross-sectional studies have also suggested that this boost in self-compassion will reduce the occurrence of NSSI in adolescents (Jiang et al., 2017). Conversely, when parents adopt negative parenting styles, such as harsh criticism, isolation, excessive identification, or emotional suppression, children cannot form a secure attachment to their parents and thus cannot develop self-compassion (Koehn and Kerns, 2018; Lathren et al., 2020).

Although previous studies have investigated the effect of parenting, attachment to parents, and self-compassion on NSSI, most were cross-sectional large sample surveys and relatively few were case–control studies. Compared to cross-sectional surveys, case–control studies have several advantages. In case–control studies, NSSI behavior can be clearly and thoroughly identified through clinical interviews, and thus a clear distinction between the NSSI group and control group can be identified. Clearly defined inclusion and exclusion criteria make it possible to investigate the risk factors associated with a specific medical condition, such as NSSI in the prior year. Through clinical interviews, we can exclude self-injury resulting from other diseases, such as rigid behaviors associated with autism spectrum disorder or self-injury with suicidal intent (Woodward, 2013). A comorbid present psychiatric diagnosis can also be identified in a case–control study.

NSSI behaviors are usually maintained over time since they have interpersonal or intrapersonal functions (Taylor et al., 2018). Clinicians hope to identify factors that influence adolescents’ motivation to stop NSSI so that they can then develop psychological treatment programs for this population. Currently, there are few studies on the factors that affect the recovery motivation of NSSI treatment. Previous research conducted by Brianna J et al. explored the factors influencing adolescents’ willingness to stop self-injury; these factors included hope, social support, coping style, and severity of psychopathological symptoms (Turner et al., 2014). However, related research remains limited. Given that attachment to parents, parenting behaviors, and self-compassion are important factors influencing adolescent NSSI, we want to understand whether these factors impact motivation to recover.

We thus conducted a case–control study for adolescents who had exhibited NSSI behaviors at least once in the last year and those who had never engaged in NSSI to study the effects of parenting style, attachment to parents, and self-compassion on NSSI behavior. At the same time, we explored the impact of these three factors on adolescents’ motivation to stop this behavior. We hypothesized that negative parenting styles and negative self-compassion would be risk factors for NSSI behaviors, and thus that they would negatively predict motivation for recovery from NSSI behaviors. Conversely, we predicted that positive parenting styles, attachment to parents, and positive self-compassion would be protective factors against NSSI behaviors and thereby positively predict motivation for recovery from such behaviors.

## Methods

### Participants

The inclusion criteria for the NSSI group were: (1) adolescents who had engaged in NSSI behavior at least once in the past year; and (2) adolescents 12–18 years old of any gender. The exclusion criteria for the NSSI group were as follows: (1) diagnosed with an organic mental health disorder, substance abuse, schizophrenia or one of its spectrum disorders, intellectual disability, or pervasive developmental disorder at present or in the past; or (2) at present has difficulty with reading or language comprehension and hence cannot participate fully with the study.

The inclusion criteria for the control group were: (1) had never engaged in any self-injury behaviors in their lifetime and (2) adolescents 12–18 years old and of any gender. The exclusion criteria for the control group were as follows: (1) diagnosed with an organic mental health disorder, substance abuse, schizophrenia or one of its spectrum disorders, intellectual disability, or pervasive developmental disorder at present or in the past; or (2) at present has difficulty with reading or language comprehension and hence cannot participate fully with the study.

### Participant recruitment

All subjects were recruited from March 2022 to April 2023. The evaluators were not blinded to the groups. To recruit adolescents with NSSI, advertisements were delivered by child outpatient clinicians in Shenzhen Kangning Hospital, and parents and adolescents who were interested in the study were referred to the research group. Those participants who met the inclusion criteria (and none of the exclusion criteria) then provided written informed consent. A child psychiatrist with intermediate seniority evaluated NSSI behavior and conducted the Kiddie Schedule for Affective Disorders and Schizophrenia-Present and Lifetime Version (K-SADS-PL) interview to determine the present comorbid diagnosis, and participants were rated according to the Hamilton Depression Scale. Then, the participants filled out the self-rating psychological questionnaires. Participants received 200 RMB after the evaluation.

To recruit healthy controls who had never engaged in NSSI, advertisements were placed throughout Shenzhen Kangning Hospital, schools, and the community. Those interested in the study signed up at Shenzhen Kangning Hospital. Individuals who met the inclusion criteria (and none of the exclusion criteria) provided written informed consent to participate in the study. A child psychiatric clinician with intermediate seniority interviewed the participants to confirm that they had never engaged in NSSI behavior before and then conducted KSADS-PL and HAMD interviews. Finally, the adolescents completed the self-rating psychological questionnaires. Participants received 200 RMB after the evaluation.

### Ethics statement

The study was conducted under the Declaration of Helsinki and approved by the Shenzhen Kanning Hospital Ethnic Committee (No. 2021-K027-01-1). All participants and their guardians signed the written informed consent form and agreed on the data being published.

### Measures

#### Kiddie schedule for affective disorders and schizophrenia-present and lifetime version

This semi-structured diagnostic interview tool, which is aligned with the DSM-IV diagnostic system, was administered by trained child psychiatrists to assess current and past mental disorders in children and adolescents. The Chinese version of this tool is widely used (Sheehan et al., 2010; Xi-Yan et al., 2012).

#### Hamilton depression scale

This is a clinician-rated scale developed by Hamilton in 1960. It is the most commonly used scale in the clinical evaluation of depression. Most items use a 5-point rating scale of 0–4, while a few items use a 3-point rating scale of 0–2 (Hamilton and Psychotherapy, 1959). A total score below 7 indicates no depression, a score of 7 to 17 indicates possible mild or moderate depression, a score of 18 to 24 indicates definite mild to moderate depression, and a score above 25 indicates severe depression. The 17-item version of this scale is widely used in China and has good reliability and validity (Yi et al., 2006).

#### Ottawa self-injury inventory

The OSI is a self-report questionnaire consisting of 28 items. The first two questions were used to assess the frequency of recent and past (1, 6, 12 months, and 1 year before) occurrences of NSSI and suicidal behaviors. The inventory also evaluates other issues related to NSSI, such as the age of NSSI onset; events that trigger NSSI thoughts, methods, and functions of NSSI; addictive features of NSSI; coping strategies; and treatment seeking. It takes about 20 min to finish the inventory. The Chinese version has good validity and reliability (Zhang et al., 2015).

In the Ottawa self-injury inventory, we chose three items to evaluate the motivation for stopping NSSI. Question 20 asked, “Once you think of self-injury, did you immediately act on it?” Answers could be yes or no. If the participant answered yes, it indicated that he immediately acted on self-injury when he thought about it and did not attempt to inhibit their NSSI impulse. If they chose no, it indicated that the participant did not immediately act on their NSSI impulse. We regarded this variable as *NSSI impulse inhibition*.

Question 25 asked, “When you tried to resist self-injury behaviors, what method did you try?” The answer could be “never tried any method” or a selection of a method provided in a list. If the participant answered that they never tried, it meant that they had no intention of resisting NSSI through other methods. In contrast, if the participant answered with one or more methods, it indicated that they were willing to resist NSSI through other methods. We regarded this variable as *NSSI resistance activity.*

Question 26 asked, “When trying to resist self-harm behavior, how much motivation do you have to stop the behavior?” This was a 5-point Likert item, where a higher score indicates higher motivation to stop NSSI. A score of 0 indicates no motivation to recover at all, a score of 1 indicates “slight motivation,” a score of 2 indicates “some motivation,” a score of 3 indicates “significant motivation,” and a score of 4 indicates “very significant motivation.” We categorize scores of 0 into one group and scores from 1 to 4 into another group, and we regarded this variable as *NSSI recovery motivation*.

#### Inventory of parent and peer attachment

The IPPA is one of the most widely used measurement tools for adolescent attachment. It was developed by Armsden and Greenberg (Armsden and Greenberg, 1987). The current version is comprised of three revised subscales: mother, father, and peer attachment subscales. Each subscale contains 25 items and assesses the degree of trust, communication, and alienation between individuals and their attachment figures. The instrument is a self-report questionnaire with a 5-point Likert scale response format. The IPPA is scored by reverse scoring the negatively worded items and then summing the response value in each subscale. This scale is widely used, and the Chinese version of the IPPA has shown good reliability and validity (Rong, 2017). In our study, we only use the mother attachment and father attachment subscales.

#### Egma minn av bardndosnauppforstran

The EMBU is a rating scale for adolescents to evaluate parenting behavior. The scale was developed by Perris et al. (2010). The Chinese version was revised by Yue Dongmei et al. and consists of 11 factors. The mother parenting style includes five elements: emotional warmth, excessive interference, rejection, punishment, and partialism. Across these five elements there are 57 items. Conversely, the father’s parenting style consists of six factors: emotional warmth, punishment, excessive interference, partialism, rejection, and overprotection. There are 58 items across these six factors. The scale adopts a 4-point rating format, with higher scores indicating more frequent use of the corresponding parenting styles by the parents (Dongmei, 1993).

#### Self-compassion scale

The SCS is a self-rating scale that evaluates self-compassion. The scale was developed by Kristin Neff and included six subscales: self-kindness, self-judgment, common humanity, isolation, over-identification, and mindfulness. It is a 5-point Likert rating scale (Kristin, 2003). The Chinese version has good reliability and validity (Chen et al., 2011). A recent study showed that the scale should be divided into two independent and distinct dimensions rather than the initially developed six factors: positive self-compassion (also referred to as simply self-compassion) and negative self-compassion (also referred to as self-coldness). The positive self-compassion dimension includes the total scores of self-kindness, common humanity, and mindfulness. In contrast, the negative self-compassion dimension includes the total scores of self-judgment, isolation, and over-identification (Brenner et al., 2017). In our study, we adopt the method of using the two dimensions.

### Data analysis

Analyses were conducted with the SPSS statistical package (IBM SPSS Statistics, version 25.0). An independent sample t-test and chi-square test were used to compare the differences in gender, age, parental marital status, and current primary diagnosis between the NSSI and control groups. An independent sample t-test was used to compare the differences between the NSSI group and the control group in terms of total scores on the HAMD, EMBU, IPPA, positive self-compassion, and negative self-compassion evaluations.

For our total sample, we used whether the participant engaged in NSSI as the dependent variable. Variables with significant differences between groups were used as covariates, and independent variables were the HAMD score, EMBU-Father Parenting score, EMBU-Mother Parenting score, IPPA-Attachment to Father score, IPPA-Attachment to Mother score, SCS positive self-compassion score, and SCS negative self-compassion score. These variables were then used to build a binary logistic regression model using a stepwise method, and we calculated the odds ratio (OR) values of father’s parenting style, mother’s parenting style, attachment to father, attachment to mother, positive self-compassion, and negative self-compassion for NSSI occurrence.

For our sample of adolescents with NSSI, we separately used NSSI impulse inhibition (0 = no inhibition; 1 = inhibition), NSSI resistance activity (0 = no resistance activity; 1 = one or more resistance activity), and NSSI recovery motivation (0 = no motivation, 1 = at least slight motivation) as dependent variables to build three different binary logistic models using a stepwise method. Independent variables were the HAMD score, EMBU-Father Parenting score, EMBU-Mother Parenting score, IPPA-Attachment to Father score, IPPA-Attachment to Mother score, SCS positive self-compassion score, and SCS negative self-compassion score. Thus, we calculated the OR values of father’s parenting style, mother’s parenting style, attachment to father, attachment to mother, positive self-compassion, and negative self-compassion in NSSI impulse inhibition, NSSI resistance activity, and NSSI recovery motivation.

## Results

### Demographics

The study included 132 adolescents who had engaged in NSSI behavior in the previous year. The NSSI frequency over the past month, past 6 months, last year, and 1 year before is presented in Table 1. Seventy-two adolescents were recruited for our non-NSSI control group. The age difference (*t* = 1.151, *p* = 0.251) was insignificant, while the differences in present primary psychiatric diagnosis (*X*^2^ = 143.33, *p* < 0.001), parental marital state (*X*^2^ = 6.162, *p* = 0.046), and gender (*X*^2^ = 10.411, *p* < 0.002) were significant (see Table 1).

**Table 1:** Demographics and NSSI frequency of the adolescents across the two groups.

	NSSI+ group (*n* = 132)	NSSI-group (*n* = 72)	*t*/*X*^2^	*P*
Mean age (m ± SD)	14.57 ± 1.49	14.31 ± 1.71	1.151	0.251
**Gender**				
Boy (*n*, %)	27 (20.5%)	30 (41.7%)	10.411	0.002
Girl (*n*, %)	105 (79.5%)	42 (58.3%)		
**Parent marital state**				
Divorced (*n*, %)	83 (62.9%)	52 (72.2%)	6.162	0.046
Married (*n*, %)	13 (9.8%)	11 (15.2%)		
Not reported (*n*, %)	36 (27.2%)	9 (12.%)		
**Present primary psychiatric diagnosis**				
Major Depression Disorder (*n*, %)	95 (71.96%)	4 (5.5%)	143.328	< 0.001
Bipolar disorder (*n*, %)	23 (17.4%)	0 (0%)		
Generalized anxiety disorder (*n*, %)	2 (1.5%)	1 (1.3%)		
Attention deficiency hyperactivity disorder (*n*, %)	2 (1.5%)	1 (1.3%)		
None (*n*, %)	10 (7.5%)	66 (91.7%)		
**NSSI frequency in the last month**				
Never (*n*, %)	9 (6.8%)	72 (100%)		
At least once (*n*, %)	41 (31.1%)			
Once a week (*n*, %)	47 (35.6%)			
Every day (*n*, %)	33 (25%)			
Not reported (*n*, %)	2 (1.6%)			
**NSSI frequency in the past 6 months**				
Never (*n*, %)	2 (1.5%)	72 (100%)		
1–5 times (*n*, %)	39 (29.5%)			
Once a month (*n*, %)	23 (17.4%)			
Once a week (*n*, %)	37 (28.0%)			
Every day (*n*, %)	30 (22.7%)			
Not reported (*n*, %)	1 (0.8%)			
**NSSI frequency in the past 6 months**				
Never (*n*, %)	5 (3.8%)	72 (100%)		
1–5 times (*n*, %)	30 (22.7%)			
Once a month (*n*, %)	37 (28.0%)			
Once a week (*n*, %)	29 (22.0%)			
Every Day (*n*, %)	28 (21.2%)			
Not reported (*n*, %)	3 (2.3%)			
**NSSI frequency: 1 year before**				
Never (*n*, %)	24 (18.2%)	72 (100%)		
1–5times (*n*, %)	38 (28.8%)			
Once a month (*n*, %)	24 (18.2%)			
Once a week (*n*, %)	23 (17.4%)			
Every Day (*n*, %)	18 (13.6%)			
Not reported (*n*, %)	5 (3.8%)			

HAMD (*t* = 16.532, *p* < 0.001), EMBU-father punishment (*t* = 5.093, p < 0.001), EMBU-father excessive interference (*t* = 4.664, *p* < 0.001), EMBU-father rejection (*t* = 3.999, *p* < 0.001), EMBU-father over-protection (*t* = 5.270, *p* < 0.001), EMBU-mother excessive interference (*t* = 5.343, *p* < 0.001), EMBU-mother rejection (*t* = 5.702, *p* < 0.001), EMBU-mother punishment (*t* = 6.770, *p* < 0.001), and SCS negative self-compassion (*t* = 9.522, *p* < 0.001) scores in adolescents with NSSI behavior were significantly higher than those in adolescents without NSSI. Meanwhile, scores in EMBU-father emotional warmth (*t* = −6.471, *p* < 0.001), EMBU-mother emotional warmth (*t* = −6.434, *p* < 0.001), IPPA-attachment to father (*t* = −5.748, *p* < 0.001), IPPA-attachment to mother (*t* = −7.583, *p* < 0.001), and SCS positive self-compassion (*t* = −7.655, *p* < 0.001) in adolescents with NSSI were significantly lower than in adolescents without NSSI (see Table 2).

**Table 2:** Comparison of adolescents with and without NSSI behavior via psychological measures.

	NSSI + Group	NSSI-group	*T*	*P*
Hamilton depressive rating scale	20.45 ± 7.42	4.48 ± 4.67	16.532	<0.001^***^
**EMBU-father parenting style**				
Emotional warmth	42.11 ± 10.76	52.24 ± 10.40	−6.471	<0.001^***^
Punishment	22.65 ± 9.07	16.62 ± 5.56	5.093	<0.001^***^
Excessive interference	23.11 ± 6.75	18.79 ± 5.34	4.664	<0.001^***^
Partialism	8.64 ± 2.93	8.44 ± 3.18	0.441	0.660
Rejection	12.18 ± 4.63	9.63 ± 3.74	3.999	<0.001^***^
Overprotection	13.68 ± 4.15	10.71 ± 3.10	5.270	<0.001^***^
**EMBU-mother parenting style**				
Emotional warmth	44.18 ± 11.94	54.73 ± 9.46	−6.434	<0.001^***^
Excessive interference	39.79 ± 10.06	32.20 ± 8.84	5.343	<0.001^***^
Rejection	16.93 ± 6.31	12.13 ± 4.42	5.702	<0.001^***^
Punishment	17.13 ± 7.03	11.21 ± 2.96	6.770	<0.001^***^
Partialism	9.08 ± 3.21	8.66 ± 3.18	0.887	0.376
**IPPA scale**				
IPPA-attachment to father	56.19 ± 18.53	71.72 ± 18.26	−5.748	<0.001^***^
IPPA attachment to mother	58.97 ± 18.79	78.48 ± 15.04	−7.583	<0.001^***^
**Self-compassion scale**				
Positive self-compassion	33.69 ± 9.99	44.42 ± 8.71	−7.655	<0.001^***^
Negative self-compassion	46.66 ± 10.53	32.55 ± 9.28	9.522	<0.001^***^

***p < 0.001.

Using whether the participants engaged in NSSI as the dependent variable, we then built a binary logistic model using a stepwise method while employing HAMD, the EMBU-Father Parenting subscale, the EMBU-Mother Parenting subscale, the IPPA-Attachment to Father total score, the IPPA-Attachment to Mother total score, SCS positive self-compassion, and SCS negative self-compassion scores as independent variables. Gender, parental marital state, and present primary psychiatric diagnosis were used as covariates. We found that the HAMD score (*p* = 0.011, OR = 1.225), SCS positive self-compassion score (*p* = 0.033, OR = 0.902), and EMBU-Mother punishment subscale (*p* = 0.031, OR = 1.278) could be included in the model. The HAMD and EMBU-Mother punishment subscale scores positively predicted the occurrence of NSSI, while SCS positive self-compassion scores negatively predicted the occurrence of NSSI (see Table 3).

**Table 3:** Binary logistic regression model investigating the effect of depressive symptoms, parenting style, attachment to parents, and self-compassion on NSSI.

	*B*	Wald	*P*	OR	95% CI	
					Lower limit	Upper limit
HAMD	0.203	6.388	0.011^*^	1.225	1.047	1.435
EMBU-mother punishment subscale	0.245	4.661	0.031^*^	1.278	1.023	1.597
Positive self-compassion	−0.104	4.552	0.033^*^	0.902	0.820	0.992

We used gender, parental marital state, and the present primary diagnosis as covariates. *p < 0.05.

Among the sample of adolescents with NSSI, using NSSI impulse inhibition as the dependent variable, we built a binary logistic model using a stepwise method while employing as independent variables HAMD, EMBU-Father Parenting, EMBU-Mother Parenting, IPPA-Attachment to Father, IPPA-Attachment to Mother, SCS positive self-compassion, and SCS negative self-compassion scores. Only the SCS positive self-compassion score could be included in the model (*p* = 0.002, OR = 1.068). Positive self-compassion positively predicted NSSI urgency inhibition (see Table 4).

**Table 4:** Binary logistic regression model for NSSI impulse inhibition.

	*B*	Wald	*P*	OR	95% CI	
					Lower limit	Upper limit
Positive self-compassion	0.66	9.951	0.002*	1.068	1.025	1.112

^*^p < 0.05.

Among the sample of NSSI participants, we took NSSI resistance activity as the dependent variable and built a binary logistic model using a stepwise method while employing as independent variables HAMD, EMBU-Father Parenting, EMBU-Mother Parenting, IPPA-Attachment to Father, IPPA-Attachment to Mother, SCS positive self-compassion, and SCS negative self-compassion scores. Gender, parental marital state, and present primary psychiatric diagnosis were used as covariates. Only SCS positive self-compassion (*p* = 0.002, OR = 1.084) and the EMBU-Father Emotional Warmth subscale (*p* = 0.019, OR = 0.955) could be included in the model (see Table 5). Positive self-compassion positively predicted NSSI resistance activity, while father emotional warmth negatively predicted it (Table 5).

**Table 5:** Binary logistic regression model for NSSI resistance activity.

	*B*	Wald	*P*	OR	95% CI	
					Lower limit	Upper limit
Positive self-compassion	0.081	9.936	0.002^*^	1.084	1.030	1.141
EMBU-father emotional warmth	−0.046	5.492	0.019^*^	0.955	0.919	0.992

*p < 0.05.

Among the sample of NSSI participants, using NSSI recovery motivation as the dependent variable, we took HAMD, EMBU-Father Parenting, EMBU-Mother Parenting, IPPA-Attachment to Father, IPPA-Attachment to Mother, SCS positive self-compassion score, SCS negative self-compassion scores as dependent variables in order to build a binary logistic model using a stepwise method. Gender, parental marital state, and present primary psychiatric diagnoses as covariates. Only SCS positive self-compassion (*p* = 0.022, OR = 1.059) could be included in the model. Positive self-compassion positively predicted NSSI recovery motivation (see Table 6).

**Table 6:** Binary logistic regression model for NSSI recovery motivation.

	*B*	Wald	*P*	OR	95% CI	
					Lower limit	Upper limit
Positive Self-compassion	0.057	5.225	0.022^*^	1.059	1.008	1.112

^*^p < 0.05.

## Discussion

Our results show that current depressive symptoms and mother-punitive parenting are risk factors for NSSI in adolescents. High levels of individual positive self-compassion were a protective factor. In addition, higher levels of positive self-compassion positively predict NSSI impulse inhibition, NSSI resistance activity, and NSSI recovery motivation. However, we unexpectedly found that the father’s emotional warmth negatively predicts NSSI resistance activity.

### Effects of punitive parenting and self-compassion on NSSI occurrence in adolescents

In our study, we found that punitive parenting was a risk factor for adolescents with NSSI. The punishment referred to here is excessive and beyond what is deserved, including physical and psychological forms of punishment. Corporal punishment can cause impairment in children’s inhibitory control abilities (Xing et al., 2018) and poorer emotional regulation abilities in the future (Wang et al., 2021). In the past year, published systematic reviews suggest that corporal punishment is associated with more severe externalized behavior problems (Avezum et al., 2023). A two-year follow-up study conducted in eight countries indicated that higher levels of maternal corporal punishment are associated with more severe anxiety and aggressive behaviors in children in the future (Lansford et al., 2014). In adolescents with NSSI, a previous study found that punitive parenting styles by mothers are a risk factor for the addictive features of non-suicidal self-harming behaviors (Zhu et al., 2022). The conclusion of the present study is consistent with that of previous research (Lansford et al., 2014; Zhu et al., 2022) thus suggesting that maternal punitive parenting could increase the risk of NSSI in adolescents.

Cross-cultural studies have found that the frequency of corporal punishment reported by mothers in China is lower than that in other countries (Lansford et al., 2005). The impact of punitive parenting practices on children’s behavioral problems varies across different cultural backgrounds. In countries where corporal punishment is commonly used, its impact on children’s behavioral problems is smaller, while in countries where corporal punishment is less common, its impact on children’s behavioral problems is more pronounced (Lansford et al., 2005). As a result, in Chinese families, it is particularly important to pay attention to the impact of corporal punishment on non-suicidal self-injury behaviors among adolescents.

Our study also found that positive self-compassion is a protective factor of adolescent NSSI. A meta-analysis of seven studies found a small effect size for self-compassion among NSSI participants, but the heterogeneity was high (Suh and Jeong, 2021). A previous study showed that the total score on the SCS scale is a mediator between the effect of negative life events and NSSI (Hasking et al., 2019). Moreover, higher total scores on the SCS scale could mitigate the effect of childhood maltreatment (Wu et al., 2023), peer victimization (Jiang et al., 2016), low peer acceptance, and behavior impulsivity (Wu et al., 2019) on NSSI. Additionally, some exercises, such as yoga, can cultivate self-compassion and reduce the occurrence of non-suicidal self-injury (Muehlenkamp and Wagner, 2022). These studies analyzed the scores on the self-compassion scale as a single factor. However, more recent studies have found that dividing the self-compassion scale into two dimensions is more accurate. These two dimensions are positive self-compassion (also referred to as self-compassion) and negative self-compassion (also referred to as self-coldness). These two factors are independent and distinct. Self-coldness cannot be regarded as a subcomponent of self-compassion, and it is recommended to analyze it separately (Brenner et al., 2017). Additionally, further research has revealed that positive self-compassion serves as a protective factor against symptoms of mental illness. Conversely, negative self-compassion is significantly more associated with mental health issues than positive self-compassion. Not dividing the SCS and using a total score might lead to an inflated association with symptoms of psychopathology (Muris and Petrocchi, 2017). When analyzing the two separate dimensions of self-compassion, NSSI behavior is associated with more severe negative self-compassion and lower levels of positive self-compassion (Jiang et al., 2017; Per et al., 2022; Liu et al., 2023). Our study reveals that positive self-compassion can be an independent factor that protects against NSSI in adolescents. This finding was consistent with previous research results (Jiang et al., 2017; Per et al., 2022; Liu et al., 2023).

### Positive self-compassion motivates NSSI recovery in adolescents

Previous studies have explored the psychological characteristics of individuals who have stopped NSSI behavior and found that individuals who have done so have fewer anxiety and depression symptoms compared to individuals who continue to self-injure (Kim and Hur, 2023). Meanwhile, externalized behavioral problems, peer bullying, alcohol, and other substance use are significantly reduced in these individuals and they have a certain sense of control over their environment (Turner et al., 2022). They have better self-efficacy, self-esteem, resilience, emotion regulation ability, and family support (Mummé et al., 2017). However, these studies did not explore the factors promoting recovery motivation when adolescents begin to receive treatment. Understanding what factors would increase the motivation for adolescents to resist NSSI urges is essential since an individual believes that they will immediately benefit from NSSI behavior (for some interpersonal or intrapersonal goal) and therefore the behavior is maintained (Taylor et al., 2018). Our research reveals that an increase in positive self-compassion scores can predict stronger recovery motivation, including refraining from immediately engaging in self-injury when self-injury thoughts arise, attempting alternative ways to resist NSSI impulses, and having an overall stronger motivation to stop NSSI.

There is a rich history of research on the relationship between self-compassion and behaviors promoting mental and physical well-being. A recent study by Phillips and Hine summarized the relationship between individual levels of self-compassion and health outcomes, incorporating 94 articles with a total of 29,588 participants. They found that higher levels of self-compassion were associated with better physical health and health-related behaviors. Additionally, interventions based on self-compassion were found to increase health-related behaviors (Phillips and Hine, 2021). Higher levels of self-compassion can promote an increase in intrinsic motivation, leading to improved mental health. Through self-compassion, individuals can also increase their motivation to seek professional help (Dschaak et al., 2021).

Meanwhile, self-compassion can also foster individuals’ positive motivation in areas beyond health behavior. For instance, individuals with more self-compassion are more likely to be aware of their weaknesses, have greater motivation to rectify and avoid past mistakes, challenge previously failed tasks, and report stronger motivation to improve upon their weaknesses (Breines and Chen, 2012). Research has found that a higher level of self-compassion in young populations can promote academic motivation among adolescents (Kotera et al., 2023) and college students (Kotera et al., 2022). Our research is consistent with past studies, showing that positive self-compassion might play an important role in adolescent NSSI recovery. Additionally, we found that positive self-compassion (as measured by the sum of scores from the three factors of self-kindness, mindfulness, and common humanity) significantly impacts NSSI recovery; meanwhile, the effects of negative self-compassion (measured by self-criticism, over-identification, and isolation) were insignificant. This suggests that positive self-compassion is a more crucial psychological resource.

The psychologist Krist Neff introduced the concept of self-compassion (Neff, 2003).

The ancient arts of loving-kindness and compassion not only provide deep spiritual insights but, as evidenced by clinical research, have been shown to elevate self-compassion scores in individuals with PTSD through the practice of loving-kindness meditation (Kearney et al., 2013). Engaging in loving-kindness meditation has been demonstrated to enhance self-kindness in patients exhibiting traits of borderline personality disorder (Feliu-Soler et al., 2017). Furthermore, when employed by individuals prone to self-criticism, traditional loving-kindness meditation can effectively diminish self-critical tendencies while concurrently boosting self-compassion scores (Shahar et al., 2015); currently, there are contemporary courses developed for self-compassion, such as compassion-focused therapy (CFT) (Gilbert, 2020) and mindful self-compassion (MSC) (Neff and Germer, 2013). In China, could it be acceptable and beneficial to incorporate the traditional, classical loving-kindness meditation or compassion meditation practices to cultivate positive recovery motivation among adolescents engaging in NSSI behaviors?

Surprisingly, we discovered that paternal emotional warmth and understanding might deter individuals from resorting to alternative methods to cope with NSSI behaviors. Prior studies recognize the significance of paternal emotional warmth and understanding in enhancing mental health among adolescents. Such warmth and understanding can diminish emotional symptoms in adolescents by enhancing their emotion regulation skills (Boullion et al., 2023). Furthermore, improved parental emotional warmth enhances adolescents’ adaptive capabilities (Lan, 2022; Gniewosz et al., 2023) while fostering compassion in offspring (Hintsanen et al., 2019). The unexpected findings in our research may be attributed to several factors. First, there might be negative parenting behaviors related to the father’s emotional warmth and understanding, such as parental indulgence (Cui et al., 2023); parental indulgence, in particular, has been linked to adverse effects on children’s adaptive capabilities (Chen et al., 2000). However, these were not measured in our study. Second, other factors may influence the relationship between fathers’ emotional warmth and understanding and adolescents’ motivation to engage in NSSI, such as adolescents’ own emotional awareness. Research has found that in Chinese adolescents, there is only a significant correlation between fathers’ emotional warmth and understanding and adolescents’ adaptive behaviors when the adolescents have high emotional awareness (Lan, 2022). Finally, our study was a cross-sectional study and thus could not establish causality. It is very likely that for adolescents lacking recovery motivation, parents may adopt a gentler, warmer, and more accepting parenting approach. Higher levels of fathers’ emotional warmth and understanding could be a result of adolescents’ lack of motivation for rehabilitation. In addition, the OR for paternal emotional warmth and understanding was 0.995, which was very close to 1, thereby suggesting that increasing the sample size might yield different results.

### Limitations

Our study has several limitations. First, there was a lack of matching in present psychiatric diagnoses between groups with NSSI and those without; these recruitment differences may potentially obscure the results. We addressed this by using these diagnoses as covariates in our analyses. Second, given that this a case–control study without longitudinal follow-up, we cannot explore the causal relationship between the identified factors and NSSI among adolescents. Furthermore, our study included patients who had engaged in NSSI at least once in the past year. In contrast, the diagnostic criteria for NSSI proposed by the DSM-5 requires at least 5 days of NSSI within a year. Therefore, the participants included in this study may have had less severe self-injurious behaviors. Additionally, this study only included data from children and it lacks information collected from the parents’ perspective; this thus casts some doubt on the data quality. There may be other unmeasured protective factors in the study, and the potential interactive effects of different factors were not measured. Therefore, further discussion is needed to understand the relationships between variables. Lastly, the study’s sample size is limited.

In the future, we need to utilize cases that are better matched for diagnosis or conduct cohort studies to understand the causal relationships between relevant factors and the condition. For instance, we can select cases that meet the NSSI diagnostic criteria proposed by the DSM-5. Additionally, we can assess the influence of other factors on NSSI such as social support, peer interaction, and disease severity. Qualitative research might unveil potential factors linked to recovery motivation. Furthermore, implementing intervention studies with longitudinal follow-up could investigate whether interventions that increase self-compassion, such as MSC, CFT, or traditional loving-kindness meditation, would facilitate NSSI recovery.

## Conclusion

This study is the first to have explored the relationship between levels of positive self-compassion and the recovery motivation of adolescents currently engaging in NSSI behavior. We found that a high level of positive self-compassion predicts increased the motivation to recover from NSSI. Additionally, current depressive symptoms and mother-punitive parenting are risk factors for NSSI in adolescents, while high levels of individual self-compassion are a protective factor. This could aid clinicians in developing preventive interventions and psychotherapeutic measures for adolescents at the beginning of NSSI treatment.

## Data availability statement

The raw data supporting the conclusions of this article will be made available by the authors, without undue reservation.

## Ethics statement

The studies involving humans were approved by Shenzhen Kangning Hospital Ethic Committee. The studies were conducted in accordance with the local legislation and institutional requirements. Written informed consent for participation in this study was provided by the participants’ legal guardians/next of kin.

## Author contributions

PL: Conceptualization, Data curation, Formal analysis, Funding acquisition, Investigation, Methodology, Resources, Validation, Writing – original draft, Writing – review & editing. JLiu: Conceptualization, Data curation, Funding acquisition, Supervision, Validation, Writing – review & editing. YL: Data curation, Writing – review & editing. JLu: Conceptualization, Funding acquisition, Supervision, Writing – review & editing.
